# On the Antagonistic Effects of Opium and Sulphate of Quinia

**Published:** 1861-08

**Authors:** Nelson Nivison

**Affiliations:** Hector, Schuyler County, New York


### ON THE ANTAGONISTIC EFFECTS OF OPIUM AND SULPHATE OF QUINIA.

BY NELSON NIVISON. M. D., of Hector, Schuyler County, New York.

That opium and quinia, when administered "simultaneously" as remedies, react upon each other, and to some extent countervail each other, is a fact which appears recently to have arrested the attention of numerous observers. Though we are prepared to admit that in a qualified sense they are "antidotes to each other," each neutralizing the *bad effects* of the other, we cannot subscribe to the rule lately laid down by Dr. Gubler, that "they ought not to be administered simultaneously." *

We claim, on the contrary, that it is precisely in virtue of the fact that these remedies do react upon each other when simultaneously administered, that the happiest therapeutic results are often produced, differing essentially from the action of either.

So true is this, so important the principle involved, so extensive its range of application, so certain and satisfactory the results, that it is believed that few of the recent improvements in practical therapeutics, have added more largely to our resources than that derived from the *combined* action of these remedies.

At the present time, perhaps no two remedies enter more largely into the general therapeutics of this country than the preparations of bark and opium. There is scarcely a disease in which their virtues have not been tested, often by many and able observers. Some of their properties have been determied with a certainty that amounts to absolute demonstration, others have now won the confidence of a majority of the profession, while other claims put forth with equal assurance have as yet failed to secure that confidence which a frequent use would necessarily imply.

Under such circumstances, any investigation which promises clearer light in regard to the properties and remedial effects of agents so powerful as these, can but be a matter of interest

* Journal des Connaissances Med. et Pharmaceutique, Aug. 30, 1858.

not alone to the medical philosopher, but especially to those who assume the practical duties of the healing art.

Before proceeding to the discussion of the conjoint action of opium and quinine, it may be well to notice some of the phenomena produced by their *separate* action.

Perhaps we cannot give the general and more obvious effects of opium better than to quote Dr. Gubler. He says: "Opium carried into the circulation induces a particular excitement, gives volume to the pulse, exalts the temperature, augments the capillary injection of the skin, and excites diaphoresis." To this we may add that while operating thus on all parts of the system it is sometimes directed with peculiar force to the brain, often producing intoxication or delirium. This is usually followed by an agreeable mental placidity, and soon all consciousness is lost in sleep.

With Dr. Gubler, we will say of quinia that its effects on the circulation are inversely to those of opium. It gives tone, or contractility to the capillaries, and thus overcomes congestion. It is anti-periodic and sedative. As incidents of its operation, we have various phenomena which indicate that it often produces great disturbance of the nervous centres.

But no mere outline of this character will give to the inquiring student an adequate idea of either the *modus operandi*, the physiological, or therapeutic effects of these articles.

'Tis the latter only which, on the present occasion, chiefly interest us. And it is only by studying their relation to certain pathological conditions that we shall be likely to arrive at a definite understanding of them.

Of their physiological effects we will simply say that both opium and quinia have a powerful affinity for the nervous system, each impressing it in a manner peculiar to itself and through its agency modifying the action of every organ in the performance of its function. Whether, as has been assumed, opium acts through the cerebro-spinal, and quinia the ganglionic system it is now our purpose to inquire.

That quinia has a powerful affinity for the nervous system we think will hardly be questioned. If any doubt should exist, the fact, we conceive, admits of demonstration. It is a law of the animal economy that no function can be performed without loss of substance. Muscular motion implies a loss of fibrin, and any activity of the brain and nervous system involves a corresponding metamorphosis of their tissues. The waste of nerve-tissue, or, in other words, the activity of the function of innervation, is easily calculated, being in direct ratio to the sum of the phosphates found in the urine.

From carefully conducted experiments it appears that quinia given in a state of health augments the amount of the phosphates, and consequently increases nervous action.

But it is in morbid states of the nervous system that the more striking effects of quinia are exhibited. Dr. Ranke (*Med. Times and Gazette*, May 30th, 1857,) while conducting a series of experiments for determining the effects of ague and quinia on the urine, ascertaiued that the paroxysm of fever greatly increased the amount of phosphoric acid. Showing most clearly that the nervous system plays an important part in the paroxysm of ague. Dr. Hammond (*Am. Jour. Med. Sci.*,) found, while experimenting on himself during an attack of intermittent, that, on the day of the first paroxysm, the amount of phosphoric acid found in the urine was 69.18. Next day (intermission), 52.95. Third day (paroxysm), 72.95. Fourth day (intermission), 55.27. On this day, quinia was administered. The next day, being the one for the paroxysm, and when the amount of phosphoric acid, calculating from the average of the preceding days, would have been 71.06, it fell to 56.22—but a trifle above that excreted on the days of intermission.

But rapid disintegration of nerve-tissues is not confined to intermittents. It is usually a prominent element in nearly all the severer forms of fever. The general debility and nonperformance of function are, doubtless, due to this cause. Continued experiment led Dr. Hammond to the conclusion that "Quinia has the power to prevent much of this great waste of nerve-material." We will add that it not only prevents destruction of nerve-tissue, but, by its well-known effects on the function of nutrition contributes greatly to the *reparative* process. It may, therefore, be justly entitled a great conservator of the nervous system in conditions of febrile excitement or nervous prostration.

But the influence of quinia is not confined to the nervous system. It has important relations to the circulation, and the character and quality of the blood. Headland believes "that its action is exerted primarily on the blood and not on the nerves." We will not discuss the question on the present occasion, nor is it necssary to our present purpose. All the vital functions are so intimately connected that no one is independent of all the rest.

The more obvious and important effects of quinia will alone be noticed; and among these, none is of so much importance, as its power of giving contractile action to the cappillaries.

This power appears to extend to all parts of the capillary system. Dr. Corrigan, Physician in Ordinary to the Queen in Ireland, says: "Quinia appears to possess the same power in giving contractility to the capillaries in the lungs which we know it possesses in so marked a degree over the capillaries and venous radicles in the spleen."

This property of quinia gives us a power over almost all forms of venous and capillary *congestion* which, perhaps, it is impossible to obtain by any other known agent.

Another effect of quinia on the circulation is that of approximating the frequency of the pulsations to the healthy standard, when much too frequent or much too slow.

We may incidentally mention that quinia enters the blood-vessels, and goes the rounds of the circulation. Tiedeman and Gmelin found it long ago in the blood of a patient to whom it had been administered, and if we needed further confirmation, we have it in the fact that from three to twelve hours after its administration it will appear in the urine. Dr. Bence Jones, M. Briquet, and many other authorities might be quoted on this point.

Quinia not only influences the circulation, but it works important changes in the character of the blood itself. We have already had occasion to notice its influence on the phosphates. Its action in diminishing the amount of uric acid is still more striking. Dr. Ranke experimented on three healthy individuals. He found that under the influence of quinia the amount of uric was reduced nearly one-half. Dr. Hammond made a series of observations during an attack of intermittent fever, where, as in all fevers, the amount of uric acid is always greatly increased. Here, likewise, the quality was promptly reduced more than half by the action of quinia.

But perhaps the most remarkable effect of quinia on the blood is the fact that it *defibrinates* it, and renders it fluid and incoaguable. This fact, mentioned by Dr. Samuel Gordon in the *Dublin Quarterly* for August, 1856, and clearly established by the experiments of Baldwin, Melier, Briquet, and other rerponsible authorities, may throw some light on its action in preventing and overcoming congestion, and subduing many forms of inflammation.

The diseases to which quinia is specially adapted usually contain an important neuropathic element. The innervation may be either deficient, irregular, or excessive; all, however, imply the existence of, or ultimately produce nervous debility. Many derangements of circulation, nutrition, secretion, sensation, and muscular motion, are included as sequelæ. An example of excessive innervation is manifest in precocious children. The vivacity, intellectual and moral development indicate a degree of nervous activity altogether disproportioned to the restorative or nutritive function. Early decay is the result.

The diseases accompanied with derangement of circulation are attended with general or local congestions. Conspicuous among them are the intermittent, remittent, continued, and pernicious fevers, and many diseases usually regarded inflammatory. Derangements of secretion, excretion, and calorification, follow in the train of disordered circulation, and imply disturbance of the ganglionic system.

Quinia is also adapted to many diseases originating in the cerebro-spinal system, as chorea, neuralgia, &c.

Of the general properties of opium, the "antagonist" of quinia, we shall speak very briefly.

One of the most important properties of opium is that of a general stimulant to the vital powers. Says Mr. Skey, the eminent Surgeon of St. Bartholomew's Hospital: "There is no drug, simple or composite, known to our pharmacologists, that possesses an equal power with opium in giving energy to the capillary system of arteries, of promoting warmth, and thus maintaining an equable balance of the circulation throughout the body,"

The property of opium, that of *equalizing the circulation*, is an invaluable one. There is scarcely a pathological condition in which it is not desirable, and without this agent sometimes difficult or impossible to obtain.

Opium not only accelerates the circulation when it is feeble, but it moderates it when excessive. This may appear paradoxical, but we think it admits of a ready solution. The circulation is subservient to innervation. While the nervous system maintains its tone, the disturbing cause must be very considerable or long continued, or the circulation will remain uninfluenced. When in an atonic condition, very slightly irritating or exciting causes will hurry the circulation. We believe a rapid pulse always implies deficient innervation. Opium is a direct stmulant of the nervous system ; it supplies for the time being the necessary power of resistance to the nervous centres, the effect of the disturbing cause is neutralized, and the circulation regains it equilibrium.

Another remarkable property of opium is the effect it has over the nutritive or reparative function. We see illustrations of this in the fact that *old ulcers* which have resisted all other means of treatment will readily heal when the system is brought under the influence of that drug. Its effects on obstinate chancres, senile gangrene, chilblains, &c., are examples in point.

A point that demands a passing notice is the general *derivative* effect of opium. By determining to the surface, internal parts are relieved of undue accumulations. In this way we can often spare the system the irritation and exhaustion that would result from counter-irritants.

We will mention still another point of great practical value, and that is the property which opium possesses of *retarding the too rapid metamorphosis of the tissues.* This enables us to maintain the integrity of the organism in exhausting fevers, wasting discharges, long-continued exposure to cold, or any protracted mental or physical suffering.

The more familiar effects of opium in removing pain, allaying irritation, procuring sleep, &c., we have not space here to notice. Enough has been written, we trust, to render it apparent that a multitude of pathological conditions demand the benefit of its influence. It is not only adapted to the inert condition of the remote vascular system, but it subdues active inflammation, and acts as a true life-sustainer when we are compelled to meet the protracted suffering incident to our frail mortality.

It is obvious that each of these "antagonistic" remedies of Dr. Gubler has a wide range of application. But the question arises: Can the several effects that we have imputed to them be confidently relied on when administered in the several pathological conditions that we have indicated? We readily admit many and various exceptions; but we claim that the number of these exceptional cases will be greatly reduced *when the two remedies are given simultaneously.*

Some of the exigencies demanding a combination of these remedies will now be indicated.

1st. There are many acute inflammatory conditions that will promptly yield to the influence of full doses of opium, where these doses cannot be given without the risk of so far paralyzing the nervous energies as to induce fatal congestions. We have seen that quinia possesses pre-eminently the power of giving contractile action to the capillaries, and thus overcoming congestion; and experience has amply demonstrated that if the proper amount of this be combined with the opium in the class of cases above referred to, as much of the latter may be administered as may be requisite to produce the effect desired.

2d. A frequent objection to the free use of opium is its tendency to so far reduce the biliary and renal secretion as to incur the risk of fatal toxæmia. Combined with quinia this tendency is to a great extent counteracted.

3d. Opium frequently reduces the respiratory action to such an extent that we are liable to have all the evils incident to imperfectly aerated blood. If, under these circumstances, we invoke the "antagonistic" effects of quinia, these unpleasant consequences are usually averted, while all the desirable effects of the opiate are retained.

4th. The *after effects* of opium are frequeutly so unpleasant as to neutralize or perhaps overbalance all the good that would otherwise result. These usually do not appear when the remedy is administered in combination with quinia.

5th. In many cases of extreme exhaustion, such as follows protracted hemorrhages and other like debilitating causes, we can temporarily arouse the energies of the system by the free use of opium; it is in fact the sheet-anchor; often the only hope. When the sensibilities of the system are thus reduced, the toleration of the drug is often truly astonishing. Under these circumstances, however, it not unfrequently happens that the quantity which is barely sufficient to produce the reaction is yet sufficient when it *does* occur to produce unpleasant narcotism. If in these cases we give quinia with the opium, we not only secure the desired action with a less amount of opium, but the more protracted operation of the quinia will enable us to maintain the reaction for any desired length of time without those frequent repetitions of the opium that would otherwise be necessary.

We may mention in this connection that persons who from accident or design have taken *overdoses* of opium and are found in a state of narcotism, are often promptly aroused by the administration of a full dose of quinia. This is especially the case in young children, who usually tolerate opiates very badly.

6th. Many patients from idiosyncrasy cannot take opium. When given in combination with quinia a large proportion of these persons take it without inconvenience.

We might multiply these examples were it necessary; but if we are thus far correct, quinia is a valuable adjuvant and often corrects the unpleasant effects of opium.

It may perhaps serve to illustrate the foregoing propositions to state that other agents than quinia, as the peculiar mental condition of lunacy, the contineed effect of cold, &c., will sometimes neutralize the bad effects of opium without impairing the good ones. Mr. Skey being obliged to ride all night in December when he had forgotten his overcoat, says: "After riding ten miles I took twenty-five drops of laudanum, and rode the remainder of the night without inconvenience." He says, it may be asked "What was the effect on the following day?" and replies, "None whatever. The cold and the opium mutually balanced each other; there could be no reaction, for the duration of the cold exceeded that of the opium."

I am inclined to think the action of the quinia in this regard analogous to that of cold. The duration of its action generally exceeds that of the opium, thus supporting the system till the effects of the opium shall have passed away.

On the other hand, certain well-known effects of *quinia* are often desirable where, from peculiarity of circumstances, we either cannot obtain them, or, doing so, we bring with them such an undesirable train of concomitauts that we are forced to dispense with the article altogether. We will notice a few of these peculiarities very briefly.

1st. The prominent feature of many diseases is dangerous *congestion* of some internal organ or organs. Here the well known action of quinia in giving contractility to the capillaries of congested parts is exceedingly desirable; but where, on trial of the remedy, we find we have imparted only a peculiar *excited* action to the general circulation, under the influence of which the congestion is aggravated rather than relieved. The addition of a sufficient amount of opium to control this excitement will not only insure the legitimate operation of the quinia on the capillaries, but by diverting the general circulation to the surface, aids still further in overcoming the congestion. In this class of cases either opium or quinia acting alone would almost certainly add to the existing congestion; *in combination* the bad effects are neutralized, and the two remedies co-operate in producing the desired relief.

2. There are many forms of *inflammation* to which, *mutatis mutandis*, the above remarks will apply with equal force. Judicious combinations of the remedies in question will often give us the most perfect control of the vital forces, and enables us to fulfil the various indications in the most satisfactory manner. There are many points of interest involved in this branch of our subject, in the discussion of which the limits of our article forbid us to enter. (For much that is valuable on the points here involved we refer to the *Dublin Hospital Gazette* for July, 1856, and the *Dublin Quarterly* for August of the same year.)

3d. There is another great class of diseases in the treatment of which quinia is almost indispensable. I allude to the idopathic fevers. Here also we are liable to have various disturbing symptoms demanding the use of opiates. Experience has demonstrated that they may be combined with quinia with the most beneficial results.

4th. There are various affections usually denominated neuralgic, which are palliated by opiates and sometimes *cured* with quinia. These cases will often yield more speedily to the *combined* influence of opium and quinia than to any other known remedies.

5th. There are numerous morbid conditions belonging to the *neuroses*, often associated with anemic states of the system where the true etiology of the disease can doubtless be traced to *defective nerve nutrition.* We have already had occasion to notice the action of both opium and quinia in modifying and improving the function of nutrition. In the present state of our knowledge there is nothing that would preclude the idea that this improved nutritive action may extend to the nerve tissue, But whatever the rational, the fact remains that very many of these neuropathic conditions will yield to the combined action of these remedies.

We are forced to the conclusion that opium and quinia are not so far "antagonistic that they should be administered simultaneously," as maintained by Dr. Gubler.—*Am. Jour. Med. Sciences.*

